# Circulating survivin levels in healthy and asthmatic pregnancy

**DOI:** 10.1186/1477-7827-12-93

**Published:** 2014-09-23

**Authors:** Andras Bikov, Renata Bocskei, Noemi Eszes, Aniko Bohacs, Gyorgy Losonczy, Janos Rigo, Ildiko Horvath, Lilla Tamasi

**Affiliations:** Department of Pulmonology, Semmelweis University, 1/C Dios arok, Budapest, H-1125 Hungary; First Department of Obstetrics and Gynecology, Semmelweis University, 27 Baross utca, Budapest, H-1085 Hungary

**Keywords:** Apoptosis, Asthma, Pregnancy, Survivin

## Abstract

**Background:**

Asthma is one of the most common conditions which complicate pregnancy. Pro- and anti-apoptotic mechanisms can be modulated by asthma accompanying pregnancy. Survivin, an anti-apoptotic protein has been implicated in the pathomechanism of asthma and also in the development of pathological pregnancies; however survivin has not been studied in pregnant asthmatics.

**Methods:**

Twenty-eight asthmatic pregnant (AP), 25 asthmatic non-pregnant (ANP), 21 healthy pregnant (HP) and 29 healthy non-pregnant (HNP) women were enrolled in this cross-sectional study. Plasma survivin concentration was determined by ELISA.

**Results:**

Plasma survivin was significantly lower in HP (1.64 /0-74.9/ pg/ml) than in HNP (24.6 /0-333.3/ pg/ml, p = 0.01). However, this difference was not observed between the asthmatic groups (p = 0.64). Similarly, there was no difference either between HNP and ANP (10.5 /0-215.4/ pg/ml, p = 0.23) or between HP and AP (13.9 /0-364.1/ pg/ml, p = 0.30) groups.

**Conclusions:**

Decreased plasma survivin levels in physiological but not in asthmatic pregnancy may suggest that the normal apoptotic mechanisms are compromised in asthmatic gestation.

## Background

Asthma is one of the most common disorders which may complicate pregnancy and it represents an increased risk for maternal and foetal complications, including preeclampsia, gestational hypertension, preterm delivery, Caesarean section, low birth weight, intrauterine growth restriction and foetal death [1, 2]. Asthma complicates 4-8% of pregnancies [2]. In addition, it is estimated that one third of asthmatic women experience asthma worsening during gestation [3]. The natural course of asthma during pregnancy is currently unpredictable due to the fact that the underlying pathophysiology is not fully elucidated. Regulation of apoptosis is a potential way to suppress immune activation during pregnancy. Pro-apoptotic mediators are released from the placenta and can be involved in the induction of increased T cell apoptosis [4, 5]. Circulating apoptotic bodies and microparticles are possible mediators for these apoptotic signals during gestation [6]. In result, an increased prevalence of apoptotic (CD95+) T cells is reported in healthy pregnant compared to healthy non-pregnant women [7]. Interestingly, recent studies reported disturbance in the pro- and anti-apoptotic balance when gestation accompanied by asthma [7, 8]; however this has not been studied in details.

Recent research focused on the role of anti-apoptotic Birc5 protein, also known as survivin, in physiological and pathological pregnancies. For brevity, we will henceforth refer to Birc5/survivin as survivin. Survivin is a member of the inhibitor-of-apoptosis family which inhibits the caspase-regulated apoptotic pathway [9]. It plays an essential role during foetal life by regulating normal cytotrophoblast development [10, 11] and survivin is highly expressed in various malignancies [12]. Its function in adult differentiated cells is not fully known, but it may regulate activation and proliferation of T cells [13].

During pregnancy survivin is produced mostly in cytotrophoblast and weakly in syncytiotrophoblast cells of the placenta [10]. It is responsible for cytotrophoblast survival by regulating cell mitosis [11]. Its role in pathological pregnancies is controversial. In hydatidiform moles and choriocarcinomas survivin levels were elevated [10, 14] while in preeclampsia decreased expression has been reported [11, 15]. Although no study has examined circulating survivin concentration in pregnancy, it is hypothesised that survivin levels are decreased as a consequence of up-regulated pro-apoptotic processes.

Survivin may also be involved in chronic inflammatory diseases such as bronchial asthma. Extracellular survivin promotes the differentiation of T cells toward Th2 line and enhances the production of some type 2 cytokines, including IL-4 and IL-13 [16]. The gene expression of survivin increases in ovalbumin-induced asthmatic mice [17, 18] and in induced sputum samples of asthmatic patients [19]. Moreover, sputum survivin mRNA levels are related to airway eosinophilia [19]. Interestingly, certain single nucleotide polymorphisms of the survivin gene are more likely associated with asthma in women [19].

However, survivin is difficult to investigate in asthmatic pregnancy as direct airway sampling methods, such as bronchial biopsy or bronchoalveolar lavage, are invasive and cannot be performed. Similarly, placental sampling also carries risk for complications. The analysis of circulating survivin is a harmless and promising method to study survivin-related processes [20–23], especially in solid tumours and leukaemia [20]. However plasma survivin has not been studied either in pregnancy or in asthma before.

As survivin is involved in asthma and pathological pregnancy, we hypothesised that it may be altered in asthmatic gestation. To investigate this, plasma survivin levels were measured in asthmatic and healthy pregnant and non-pregnant women.

## Methods

### Study subjects

Twenty-eight asthmatic pregnant (AP, 31 ± 5 years), 25 asthmatic non-pregnant (ANP, 32 ± 7 years), 21 healthy pregnant (HP, 31 ± 5 years) and 29 healthy non-pregnant (HNP, 30 ± 5 years) women were enrolled. The AP group comprised volunteers in the 2^nd^ trimester (N = 19, 20 ± 5 gestational weeks) or 3^rd^ trimester (N = 9, 34 ± 4 gestational weeks), while the HP group consisted of participants in the 2^nd^ trimester (23 ± 3 gestational weeks). All volunteers were Caucasian except for one AP women who had Asian origin.

Asthmatic patients were recruited at the outpatient clinic of Department of Pulmonology. Asthma was diagnosed by a respiratory medicine specialist according to the Global Initiative for Asthma (GINA) guidelines. Asthmatic patients with exacerbations within the last 6 months were not included. Nineteen ANP and fifteen AP subjects used inhaled corticosteroids regularly, while others were considered steroid-naive. The asthma was considered well-controlled or partially controlled in 13 ANP and 14 AP subjects and uncontrolled in 12 and 14 subjects, respectively.

Pregnant women were recruited at the First Department of Obstetrics and Gynecology. In all cases, the pregnancy and labour were uncomplicated and pregnant women gave birth to healthy children. Volunteers with twin pregnancies or in whom later preeclampsia developed were not studied. HNP volunteers were workers and students of Semmelweis University.

Subjects with any chronic disease, including hypertension, diabetes or malignancies were excluded. None of the participants were current or ex-smokers or had any respiratory tract infection within 4 weeks of the study.

### Study design

In all subjects, venous blood was collected in EDTA-tubes. In eight 2^nd^-trimester asthmatic pregnant volunteers, sample collection was repeated in the 3^rd^ trimester (in the cross-sectional analysis only the sample from the 2^nd^ trimester was used). In asthmatic subjects, additional lung function and fractional exhaled nitric oxide (FE_NO_) [24] measurements were performed and asthma control was evaluated with the Asthma Control Test (ACT) [25].

The study was approved by the Semmelweis University Ethics Committee (TUKEB 110/2007), and all patients gave written informed consent prior to participation in the study.

### Plasma survivin measurements

Plasma was separated according to the ELISA kit guidelines and stored at -80°C until survivin measurements. Plasma survivin levels were determined by a commercially available ELISA kit (DSV00, R&D Systems, Abingdon, UK). The detection limit was 4.44 pg/ml, as it was reported by the manufacturer. The mean intra-assay coefficient for variation of duplicate samples was 22%.

### Statistical analysis

We used Graphpad Prism 4.0 (GraphPad Software Inc., San Diego, CA, USA) for statistical analysis. The normality distribution of the data was assessed by Kolmogorov–Smirnov test. Plasma survivin was compared among groups with two-way ANOVA followed by Bonferroni post hoc test. Unpaired t-test was applied to compare lung function variables, steroid use and neonatal birth weight, while Mann–Whitney test was used to compare FE_NO_ and ACT levels. The relationship between survivin levels and clinical variables was analysed with Spearman tests. Pearson and Spearman tests were used to correlate clinical variables within groups. Since plasma survivin as well as FE_NO_ levels were not normally distributed, these variables were expressed as median/range/, otherwise as mean ± SD. Samples with plasma survivin levels below the detection limit were assigned to have 0 pg/ml of survivin. p < 0.05 was considered significant.

The sample size was calculated to find differences in plasma survivin levels among the four groups using an effect size of 0.35 and a statistical power (1-β) of 0.80 taking into account the asymptotic relative efficiency of non-parametric tests [26].

## Results

### Comparison of the four groups

The two asthmatic groups (AP and ANP) were comparable in terms of lung function, inhaled corticosteroid use, FE_NO_ and asthma control (all p > 0.05). Similarly, there was no difference in neonatal birth weight, week of delivery or in the 0- and 5-minute Apgar scores between the AP and HP groups (all p > 0.05, Table 1).

**Table 1:** **Clinical characteristics of study subjects**

	AP N = 28	ANP N = 25	HP N = 21	HNP N = 29	P value
*FEV*_*1*_ *; L*	2.9 ± 0.4	2.8 ± 0.7	ND	ND	0.54
*(% pred)*	(90 ± 11)	(88 ± 18)			(0.66)
*FVC; L*	3.6 ± 0.5	3.7 ± 0.8	ND	ND	0.85
*(% pred)*	(99 ± 13)	(100 ± 15)			(0.68)
*FE*_*NO*_ *; ppb*	19 (8–115)	19 (5–82)	ND	ND	0.62
*ACT*	20 (8–25)	20 (9–25)	ND	ND	0.76
*ICS; BDP eq.*	200 (0–2000)	400 (0–1000)	NA	NA	0.25
*Neonatal birth weight; g*	3548 ± 714	NA	3442 ± 320	NA	0.59
*Apgar; 1 and 5 minutes*	9 and 10	NA	9 and 10	NA	0.27 and 0.95
*Week of delivery*	39 (36–42)	NA	39 (36–41)	NA	0.44

AP: asthmatic pregnant, ANP: asthmatic non-pregnant, HP: healthy pregnant, HNP: healthy non-pregnant, ACT: asthma control test, BDP eq.: beclomethasone dipropionate equivalent, FE_NO_: fractional exhaled nitric oxide, FEV_1_: forced expiratory volume in one second, FVC: forced vital capacity, ICS: inhaled corticosteroid, NA: not applicable, ND: not determined, ppb: particles per billion. Data are expressed as mean ± SD or median (range).

### Circulating survivin levels and their relationship to clinical parameters

**Figure 1:**
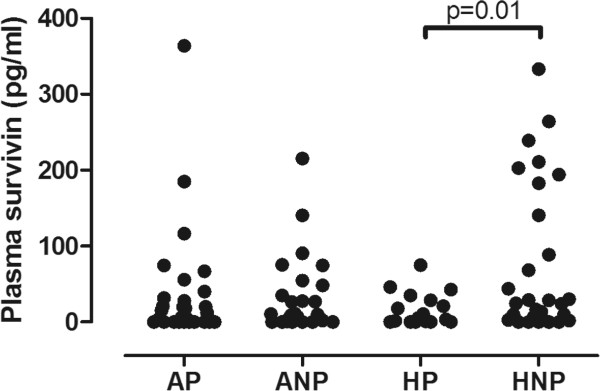
**Plasma survivin levels in the four groups.** Plasma survivin levels were significantly lower in HP subjects compared to HNP volunteers. AP: asthmatic pregnant, ANP: asthmatic non-pregnant, HP: healthy pregnant, HNP: healthy non-pregnant.

Survivin was detectable in 61% of AP, 43% of HP, 68% of ANP and 72% of HNP women. Comparing the four groups using two-way ANOVA, significantly lower plasma survivin levels were noted in pregnancy (p = 0.04), however asthma had no effect (p = 0.59). Bonferroni post hoc test revealed that pregnancy-related differences were present only in healthy groups (1.64/0-74.9 pg/ml/ vs. 24.6 /0-333.3/ pg/ml, p = 0.01, HP vs. HNP, respectively), while there was no difference when the asthmatic non-pregnant (10.5 /0-215.4/ pg/ml) and asthmatic pregnant (13.9/0-364.1/) patients were compared (p = 0.64). If the 3 outliers in the AP group were excluded the difference between the 4 groups was still significant (p < 0.01). Comparing asthmatic patients to the corresponding non-asthmatic subjects no difference was found either between pregnant (p = 0.30) or non-pregnant (p = 0.23) groups (Figure 1). When AP patients only in the 2^nd^ trimester were compared to HP volunteers the difference was still not significant (p = 0.25).

**Figure 2:**
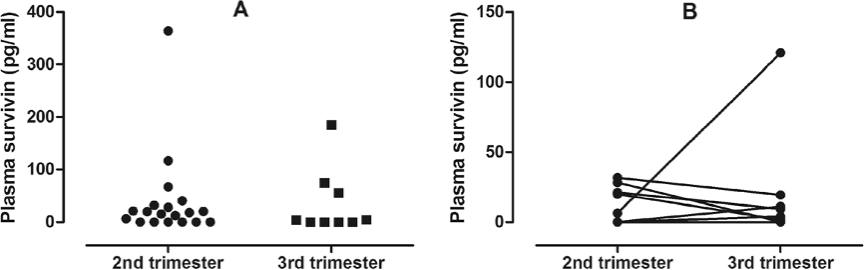
**Plasma survivin levels in the 2** ^**nd**^ **and 3** ^**rd**^ **trimesters of asthmatic pregnancy.** There was no difference in plasma survivin between the 2^nd^ and 3^rd^ trimesters either when 2^nd^ and 3^rd^ trimester asthmatic pregnant women were compared **(Panel A)** nor when the temporal changes were assessed in eight subjects **(Panel B)**.

There was no difference between AP subjects in the 2^nd^ (17.8 /0-364.1/ pg/ml) and 3^rd^ trimester (0 /0-185.0/ pg/ml, p = 0.61). Nor were the survivin levels in the same individual (8 AP subjects) different between the two time points (13.1 /0-31.6/ pg/ml vs. 6.6 /0-121.0/ pg/ml, 2^nd^ vs. 3^rd^ trimester, respectively, p = 0.79, Figure 2). This indicates that differences in gestational age between the AP and HP groups did not bias the results on survivin. Comparing survivin levels depending on newborn gender there was no difference either in AP (p = 0.47) or HP (p = 0.45) groups.

The relationships between plasma survivin levels and lung function variables, FE_NO_ or asthma control in the AP and ANP groups were not significant (all p > 0.05). Nor was there any relationship between plasma survivin and gestational weeks or neonatal birth weight in either of the AP and HP groups (all p > 0.05). Comparing asthmatic patients using inhaled corticosteroids (ICS) with steroid-naive subjects, no difference was observed in plasma survivin either in the ANP (p = 0.80) or AP groups (p = 0.58). Similarly, there was no relationship between ICS dose and plasma survivin levels either in ANP (p = 0.19) or AP (p = 0.69) subjects.

### Relationship between clinical variables

In the ANP group a significant relationship was found between FEV_1_ and ACT (r = 0.43, p = 0.03) and there was a statistical tendency for inverse correlation between FEV_1_ and FE_NO_ levels (r = -0.44, p = 0.06). Interestingly, these correlations were not present in AP subjects either when the 2^nd^ and 3^rd^ trimester groups were analysed together or separately.

## Discussion

In the current study we investigated plasma survivin levels in asthma, together with asthmatic and healthy pregnancies. We found that circulating survivin is decreased during gestation, which was blunted in asthmatic pregnancy.

This is the first study analysing circulating survivin levels in pregnancy, however the intracellular expression during gestation has already been investigated in physiological and pathological circumstances. It is known that this molecule is produced by the placenta and has an important role in the normal cytotrophoblast development [10, 11]. Its expression is tightly regulated as both increased and reduced productions are associated with pathological pregnancies [10, 11, 14, 15].

The source and function of extracellular survivin in pregnancy is not known. It may originate from dead cells, but survivin can also be actively released by living cells [27]. In malignancies, extracellular survivin is taken up by the surrounding cancer cells inhibiting their apoptosis, accelerating their proliferation and increasing their invasive potential [27]. A recent study described that survivin produced by cancer cells also inhibits T cell activation and proliferation [16]. Hence, decreased survivin levels in pregnancy may be associated with enhanced T cell activation. Supporting this, we have previously reported that non-asthmatic pregnancy is associated with activation and apoptosis of T cells [7]. Survivin affects lymphocyte subtypes in different ways. It decreases the number and suppresses the function of CD8+ T cells, skewing immunity towards the Th2 direction, but not altering the regulatory T cell and Th17 ratios [16]. In addition, IFN-γ and IL-2 levels are decreased while IL-4 and IL-13 concentrations are increased in the presence of survivin [16]. It is known that healthy pregnancy is associated with altered T cell balance [28] and cytokine profiles [29] with elevated proportions of CD8+ cells [28] and decreased levels of IL-4 [29].

Another possible reason for low extracellular survivin in pregnancy might be the reduced production of vascular endothelial growth factor (VEGF) [30]. It is known that the expression of survivin is induced by VEGF [31] which is supported by a significant relationship between plasma survivin and VEGF levels [21]. Finally, it is known that survivin is down-regulated by progesterone [32] the level of which is highly elevated during gestation. As we only investigated pregnant subjects in the 2^nd^ and 3^rd^ trimesters, we do not known if extracellular survivin is equally low during the whole course of pregnancy. Only one study measured placental survivin mRNA, showing reducing levels throughout the pregnancy [15]. We did not find significant differences in survivin levels between the 2^nd^- and 3^rd^-trimester pregnant asthmatics, however this analysis was poorly powered in the current study and we cannot rule out the possibility that survivin levels may change in non-asthmatic pregnant subjects.

Asthma may modulate pregnancy-related immune responses [28, 33]. For instance, CD8+ T cell prevalence is decreased [28], while IL-4 and IFN-γ levels are elevated [33] in asthmatic gestation. The absence of a physiological decrease in survivin levels in asthmatic pregnancy may contribute to these immunological changes. Nonetheless, IFN-γ, known to be increased in asthmatic pregnancy [33] may up-regulate survivin expression [13] contributing to its blunted decrease seen in the current study.

In addition, various studies suggest that asthma also suppresses the anti-apoptotic mechanism seen in physiological pregnancy. We have previously described that lymphocyte apoptosis is enhanced in healthy pregnancy; however this effect is limited in asthmatic pregnant women [7]. Similarly, another anti-apoptotic agent [34], heat shock protein 70 was also found to be decreased in normal pregnancy [35], but not in asthmatic pregnant women [8]. Our present results are consistent with these previous findings.

Recent studies supported the role of survivin in the pathomechanism of asthma [17–19]. We could not find any differences between asthmatic and non-asthmatic subjects either when the pregnant or non-pregnant women were compared. Similarly, there was no correlation with any of the asthma variables. This might suggest that survivin-related asthmatic processes are localised in the lungs, as in the previous human study, only airway samples were analysed [19]. In the present study, asthmatic subjects with recent exacerbation were excluded, and asthma was considered relatively stable in participants. Despite the fact that there was no association between survivin levels and clinical variables of asthma, we cannot exclude the possibility that heightened disease activity (i.e. during exacerbation) might be related with increased systemic survivin. Of note, increased survivin in induced sputum samples of asthmatic patients was noted even in stable subjects [19].

Only a few studies have examined plasma survivin to date in various diseases including solid tumours, leukaemia, rheumatoid arthritis and HCV infection [20–23]. In overall, the median values were very close to the lower limit of detection with around 30% of the samples below the limit of detection which is in line with the observations of the current study. Survivin was even more poorly detectable in pregnancy which further confirms that this molecule is decreased during gestation, but unfortunately this also limits the statistical power of our conclusions. Previous studies measured survivin in plasma samples with ELISA which is a more feasible method to analyse survivin in <100 pg/ml concentration range than Western blot which has a detection limit around 100 pg/ml in plasma samples. Two studies used the same commercially available ELISA kit as in our study [21, 23], but unfortunately neither of them reported intra-assay variability. The relatively poor analytical repeatability of this analytical method and the high proportion of samples with survivin concentration below the detection limit may not allow to draw final conclusions from some analyses done on small number of samples (i.e. the effect of gestational age or clinical outcomes of asthma or gestation). Therefore, the results on these statistics should be interpreted carefully. Of note, further studies are warranted to optimise the medium (serum, EDTA or citrate plasma) for survivin measurements, as the healthy values tended to be higher in a previous study using citrate tubes than in our results measured in samples collected in EDTA tubes [23].

## Conclusions

In summary, for the first time, we reported significantly lower levels of plasma survivin in physiological but not in asthmatic gestation. Further studies are warranted to fully investigate the influence of survivin on apoptotic and immunological processes in pregnancy.

## Abbreviations

ACT: Asthma control test
AP: Asthmatic pregnant
ANP: Asthmatic non-pregnant
BDP eq.: Beclomethasone dipropionate equivalent
FENO: Fractional exhaled nitric oxide
FEV1: Forced expiratory volume in one second
FVC: Forced vital capacity
HP: Healthy pregnant
HNP: Healthy non-pregnant
ICS: Inhaled corticosteroid
LD: Limit of detection
SD: Standard deviation
VEGF: Vascular endothelial growth factor.
